# Inhibition of caspase-1 activation in gram-negative sepsis and experimental endotoxemia

**DOI:** 10.1186/cc9974

**Published:** 2011-01-18

**Authors:** Evangelos J Giamarellos-Bourboulis, Frank L van de Veerdonk, Maria Mouktaroudi, Maria Raftogiannis, Anastasia Antonopoulou, Leo AB Joosten, Peter Pickkers, Athina Savva, Marianna Georgitsi, Jos WM van der Meer, Mihai G Netea

**Affiliations:** 1 4th Department of Internal Medicine, University of Athens, Medical School, 1 Rimini Str. 12462 Athens, Greece; 2 Department of Medicine and Nijmegen Institute for Infection, Inflammation and Immunity (N4i), Radboud University Nijmegen Medical Centre, 8 Geert Grooterplein, 6500 HB Nijimegen, The Netherlands; 3 Department of Critical Care Medicine, Radboud University Nijmegen Medical Centre, 8 Geert Grooterplein, 6500 HB Nijimegen, The Netherlands

## Abstract

**Introduction:**

Down-regulation of *ex-vivo *cytokine production is a specific feature in patients with sepsis. Cytokine downregulation was studied focusing on caspase-1 activation and conversion of pro-interleukin-1β into interleukin-1β (IL-1β).

**Methods:**

Peripheral blood mononuclear cells were isolated from a) 92 patients with sepsis mainly of Gram-negative etiology; b) 34 healthy volunteers; and c) 5 healthy individuals enrolled in an experimental endotoxemia study. Cytokine stimulation was assessed *in vitro *after stimulation with a variety of microbial stimuli.

**Results:**

Inhibition of IL-1β in sepsis was more profound than tumour necrosis factor (TNF). Down-regulation of IL-1β response could not be entirely explained by the moderate inhibition of transcription. We investigated inflammasome activation and found that in patients with sepsis, both pro-caspase-1 and activated caspase-1 were markedly decreased. Blocking caspase-1 inhibited the release of IL-1β in healthy volunteers, an effect that was lost in septic patients. Finally, urate crystals, which specifically induce the NLPR3 inflammasome activation, induced significant IL-1β production in healthy controls but not in patients with sepsis. These findings were complemented by inhibition of caspase-1 autocleavage as early as two hours after lipopolysaccharide exposure in volunteers.

**Conclusions:**

These data demonstrate that the inhibition of caspase-1 and defective IL-1 β production is an important immunological feature in sepsis.

## Introduction

Despite the increase of our knowledge on the pathophysiology of sepsis, mortality remains high [1]. A vast number of agents aiming to modulate the inflammatory response of the host have failed to provide any clinical benefit [2]. During the initiation of the inflammatory process in sepsis syndrome, microbial components such as lipopolysaccharide (LPS), muramyldipeptide (MDP), flagellin and bacterial DNA interact with pattern recognition receptors (PRRs) that are located either on the cell membrane or in the cytoplasm of host cells. Interaction of these ligands with specific PRRs leads to the activation of a series of intracellular effector molecules and ultimately to nuclear translocation of transcription factors such as of NF-κB (Nuclear Factor kappaB) and subsequent gene expression of pro-inflammatory cytokines like TNFα (tumor necrosis factor-alpha), IL(interleukin)-1β, IL-6 and IL-8 [3]. Soon after the onset of sepsis, white blood cells (monocytes and lymphocytes) of critically ill patients are severely impaired in their capacity to produce these pro-inflammatory cytokines *in vitro *[3]. This impairment is part of a second hypo-inflammatory state of the septic cascade also known as immunoparalysis. Lower expression of MHC class II and decreased lymphocyte proliferation, as well as the induction of lymphocyte apoptosis in sepsis are also part of the immunoparalysis state [4]. This latter stage of sepsis is associated with an increased risk for nosocomial infection and death.

IL-1β is a major component of the pro-inflammatory response during sepsis [5]. IL-1β is produced as an inactive pro-peptide that needs to be cleaved by the cysteine protease caspase-1 in order to become bioactive [6]. Procaspase-1 has to be converted into the active cysteine protease caspase-1, which in turn cleaves pro-IL-1β. Caspase-1 activation is mediated by the inflammasome, a multimeric protein platform that is activated after recognition of danger signals such as ATP and uric acid [7,8]. As a consequence, production of IL-1β when sepsis appears may be modulated either at the level of gene transcription or at the level of cleavage of pro-IL-1β. The aim of the present study is to define if defective production of IL-1β from monocytes in clinical sepsis is due to down-regulation of gene expression or inhibition of the inflammasome. To this end, we investigated the down regulation of IL-1β in sepsis and experimental endotoxemia in human volunteers with emphasis on the activation of caspase-1 and subsequent IL-1β production.

## Materials and methods

### Study design

This prospective study was conducted in the 4^th ^Department of Internal Medicine of ATTIKON University Hospital of Athens during the period September 2007 to September 2008. A total of 92 patients and 34 healthy volunteers were enrolled. Written informed consent was provided by the patients or their first-degree relatives if patients were unable to provide the consent. The study protocol was approved by the Ethics Committees of the ATTIKON University Hospital. Each patient was enrolled once.

Inclusion criteria were: a) age ≥ 18 years old; b) sepsis due to acute pyelonephritis or primary Gram-negative bacteremia or acute intrabdominal infection; and c) blood sampling within 24 hours from advent of signs of sepsis. Exclusion criteria were: a) HIV infection; b) neutropenia (defined as an absolute neutrophil count lower than 1,000 neutrophils/mm^3^); c) intake of corticosteroids defined as any oral dose equal to or greater than 1 mg/kg of equivalent prednisone for more than one month; d) pregnancy; e) history of any organ transplantation; and f) acute pancreatitis.

Patients with sepsis syndrome were classified as suffering from uncomplicated sepsis, severe sepsis and septic shock, according to standard definitions [9].

Acute pyelonephritis was diagnosed in every patient with all the following signs [10]: a) core temperature > 38°C or < 36°C; b) lumbar tenderness; and c) ≥ 10 WBC/high-power field of spun urine or ≥ 2+ in dipstick test for WBCs and nitrates or radiological evidence consistent with the diagnosis of acute pyelonephritis. Primary Gram-negative bacteremia was diagnosed in every patient presenting with at least one peripheral blood culture positive for Gram-negative bacteria without indication for another infection site despite thorough work-out [11]. Acute intra-abdominal infection was diagnosed in every patient presenting with all the following signs [11]: a) core temperature > 38°C or < 36°C; b) WBC count < 4,000/mm^3 ^or > 12,000/mm^3^; and c) indicative radiological evidence in abdominal computed tomography or abdominal ultrasound.

Patients were followed for 28 days. For every patient, a complete diagnostic work-out was performed comprising history, thorough physical examination, WBC count, blood biochemistry, arterial blood gas, blood cultures from peripheral veins and central lines, urine cultures, chest x-ray and chest and abdominal computed tomography or abdominal ultrasound if considered necessary.

### Endotoxemia model in healthy volunteers

The study protocol is approved by the Ethics Committee of the Radboud University Nijmegen Medical Centre and complies with the Declaration of Helsinki including current revisions and the Good Clinical Practice guidelines. Written informed consent was obtained from all study participants. Five subjects described in the present study participated in a larger similar trial [12]. U.S. reference *Escherichia coli *endotoxin (lot Ec-5, Center for Biological Evaluation and Research, Food and Drug Administration, Bethesda, MD, USA) was used. Ec-5 endotoxin, supplied as a lyophilized powder, was reconstituted in 5 ml saline 0.9% for injection and vortex mixed for at least 10 minutes after reconstitution. The endotoxin solution was administered as a single intravenous bolus injection for one minute at a dose of 2 ng/kg of body weight by one forearm vein. Patients were observed on an intensive care unit during the entire period of the study, and blood samples were collected by venipuncture at the time points indicated.

### Isolation and stimulation of PBMCs

A total of 20 ml of heparinized blood was sampled within less than 24 hours of advent of signs of sepsis by venipuncture of one forearm vein under aseptic conditions and processed within less than one hour. Blood sampling was performed in a similar way from healthy donors and just before (t = 0), two hours after (t = 2) and eight hours after (t = 8) LPS infusion.

Heparinized venous blood was layered over Ficoll Hypaque (Biochrom, Berlin, Germany) and centrifuged for 20 minutes at 1,400 g. Separated mononuclear cells (PBMCs) were washed three times with ice-cold PBS (phosphate buffered saline) (pH: 7.2) (Biochrom) and counted in a Neubauer chamber. Their viability was more than 99% as assessed by trypan blue exclusion of dead cells. They were then diluted in RPMI 1640 enriched with 2 mM of L-glutamine, 100 U/ml of penicillin G, 100 μg/ml of gentamicin and 10 mM of pyruvate and suspended in wells of a 96-well plate (Greiner, Alphen a/d Rijn, The Netherlands). The final volume per well was 200 μl with a density of 2 × 10^6 ^cells/ml.

PBMCs were stimulated with the following stimuli:

a) LPS of *Escherichia coli *O55:H5 at concentrations of 0.1 and 10 ng/ml (Sigma Co, St. Louis, MO, USA), which is a TLR4 agonist;

b) 5 μg/ml of Pam3Cys-SKKK (EMC Microcollections, Tübingen, Germany) which is a TLR2 agonist;

c) 5 μg/ml of phytohemagglutin (PHA) of *Phaselolus vulgaris *(PHA-L, Roch Diagnostics GMBH, Mannheim, Germany);

d) 5 × 10^5 ^colony-forming units (CFU)/ml of heat-killed isolates of *Candida albicans*, of *Pseudomonas aeruginosa *and of methicillin-resistant *Staphylococcus aureus *(MRSA). All are blood isolates from patients with severe sepsis killed after heating a 5 × 10^7 ^CFU/ml inoculum for six hours at 90°C. *Pseudomonas aeruginosa *isolate is already applied in former studies of our group [13]. It is multidrug-resistant to piperacillin/tazobactam, imipenem, amikacin and ciprofloxacin, as assessed after estimation of minimum inhibitory concentrations by a microdilution technique using CLSI breakpoints. Resistance to methicilln of the MRSA isolate was assessed after detection of the *mecA *gene by PCR [14]. For all three isolates heat-killing was ascertained after six serial 1:10 dilutions of the inactivated inoculum.

e) crystals of MSU at concentrations of 10 and 100 μg/ml prepared as described elsewhere [15].

Stimulations were performed in the absence and presence of 5 μmol/l of the caspase-1 inhibitor (ICE-i) Ac-Tyr-Val-Ala-Asp-2,6-dimethylbezoyloxymethylketone (YVAD). YVAD was purchased from Biomol (Plymouth Meeting, USA) and solubilized in dimethyl sulfoxide (DMSO) at 10 mg/ml.

PBMCs of healthy subjects before and during experimental endotoxemia were stimulated without/with 10 ng/ml of LPS, as described above.

After 24 hours of incubation at 37°C in 5% CO_2 _atmosphere, the plates were centrifuged. Supernatants were kept stored at -70°C until assayed.

### Cytokine measurements

Concentrations of IL-1β, IL-6 and ΤNFα were estimated in supernatants in duplicate by an enzyme immunoassay (R&D Systems, Minneapolis, MN, USA). The lower detection limits were: 20 pg/ml for IL-1β; 20 pg/ml for IL-6; and 40 pg/ml for TNFα. Concentrations of IL-10 were also determined in supernatants of LPS-stimulated PBMCs of 44 sepsis patients by an enzyme immunoassay (R&D Systems). The lower detection limit was 20 pg/ml.

### Western blot analysis for caspase-1

A total of 5 × 10^6 ^PBMCs from four healthy controls; from five patients with sepsis; and from four healthy volunteers before LPS infusion (t = 0) and two hours after infusion (t = 2) were lysed in 100 μl lysis buffer (50 mM Tris, pH 7.4, 150 mM NaCl, 2 mM EDTA, 2 mM EGTA, 10% glycerol, 1% Triton X-100, 40 mM β-glycerophosphate, 50 mM sodium fluoride, 200 μM sodium vanadate, 10 μg/ml leupeptin, 10 μg/ml aprotinin, 1 μM pepstatin A, and 1 mM phenylmethylsulfonyl fluoride) and stored at -70°C. Protein concentrations were determined by BCA protein assay (Thermo Scientific, Rockford, IL, USA) before loading on a 12% SDS-bisacrylamide gel (Bio-Rad, Hercules, CA, USA) and anti-actin-antibody (Santa Cruz Biotechnology, Santa Cruz, CA, USA). Proteins were transferred onto a nitrocellulose membrane by using an I-Blot apparatus (Invitrogen, Carlsbad, CA, USA). Rabbit-anti-caspase-P10 antibody (Santa Cruz Biotechnology) was used followed by goat-anti-rabbit-Alexa680 (LI-COR Biosciences, Lincoln, NE, USA). The protein bands were visualized by an infrared image scanner (Odyssey, Westburg, The Netherlands).

### Quantitative PCR for mRNA expression of TNFα and IL-1β

PBMCs were stimulated, as stated above, with or without 1 ng/ml of LPS. After four hours of incubation at 37°C in 5% CO_2 _and plate centrifugation, the cell pellet was lysed with 400 μl of Trizol (Invitrogen, Karlsruhe, Germany) and kept at -80°C until extraction of RNA. RNA was extracted by chloroform gradient centrifugation followed by treatment for 30 minutes at 37°C with 0.04 U/μl of DNAase (Ambion, Austin, USA). A total of 1.5 μg of RNA was applied for the production of cDNA using 0.4 mM of dNTPs (New England BioLabs, Ipswitch, MA, USA), 1 U of RNA-sin (New England BioLabs), 10 mM DTT (New England BioLabs) and 5× of the reverse transcriptase buffer in a Mastercycler 5330 apparatus using appropriate blanks (Eppendorf, Antisel, Athens, Greece). After an initial incubation step of 10 minutes at 65°C, 1 μU of reverse transcriptase (New England BioLabs) was added followed by three steps: 10 minutes at 25°C; 50 minutes at 42°C; and 15 minutes at 70°C. cDNA was kept at -80°C until assayed.

Expression of mRNA was tested by the iCycler system (BioRad) using per reaction tube 1 μl of cDNA, 0.1 mg/ml of sense and antisense primers, 3 mM of MgCl_2 _(New England BioLabs), 0.25 mM of dNTPs (New England BioLabs), 10× buffer and 1 mM of *Taq *polymerase with SYBR-Gr as a fluorochrome. Primer sequences were: for TNFα sense 5'-TGG CCC AGG CAG TCA GA-3' and antisense 5'-GGT TTG CTA CAA CAT GGG CTA CA-3'; for IL-1β sense 5'-GCC CTA AAC AGA TGA AGT GCT C-3' and antisense 5'-GAA CCA GCA TCT TCC TCA G-3'; and for β_2_-microglobulin sense 5'-ATG AGT ATG CCT GCC GTG TG-3' and antisense 5'-CCA AAT GCG GCA TCT TCA AAC-3'. After an initial denaturation step for 10 minutes at 95°C, 34 cycles were performed. Each cycle consisted of three steps; denaturation for 30 seconds at 95°C; annealing for 30 seconds at 72°C; and elongation for 30 seconds at 95°C. Amplification was followed by a melting curve; appropriate blanks were applied. The PCR product was recognized after 3% agarose gel electrophoresis and ethidium bromide staining. Quantitative results were expressed as defined by the PFAFFL equation [15] using the efficiency of a standard curve created with known cDNA.

### Statistical analysis

Results were expressed as means ± SE. Distribution of cytokine concentrations after stimulation within the healthy control group, the uncomplicated sepsis group, the severe sepsis group and the septic shock group was normal; comparisons between groups were done by ANOVA with post hoc analysis by Bonferroni to avoid any random correlation. Comparisons of a) mRNA transcripts; and b) cytokine release after stimulation with both LPS and MSU between controls and patients were done by the Mann-Whitney U test. Comparisons of a) cytokine release before and after treatment with the YVAD inhibitor; and b) cytokine release before and after treatment with MSU were done by the Wilcoxon' s signed rank test. *P*-values below 0.05 were considered significant.

## Results

### Study population

Demographic and clinical characteristics of the 92 septic patients are shown in Table 1. Forty-nine patients were classified as uncomplicated sepsis, 26 as severe sepsis and 17 as septic shock. Of the 34 healthy donors, 18 were male and 14 female; their mean age was 33.20 ± 5.51 years (mean ± SD).

**Table 1:** Demographic and clinical characteristics of the 92 septic patients enrolled in the study.

Gender (male/female)	48/44
Age (years, mean ± SD)	65.59 ± 19.88
APACHE II score (mean ± SD)	14.36 ± 7.48
Sepsis stage (number, %)	
Uncomplicated sepsis	49 (53.3)
Severe sepsis	26 (28.3)
Septic shock	17 (18.5)
Underlying infection (number, %)	
Acute pyelonephritis	38 (41.3)
Primary bacteremia	27 (29.3)
Acute intrabdominal infections	27 (29.3)
Co-morbidities (number, %)	
Diabetes melliitus type 2	15 (16.3)
Chronic obstructive pulmonary disease	6 (6.5)
Chronic renal failure	9 (9.8)
Chronic heart failure	7 (7.6)
Implicated pathogen* (number, %)	
*Escherichia coli*	21 (22.8)
*Klebsiella pneumoniae*	11 (11.9)
Other Gram-negatives	11 (11.9)
Death (number, %)	22 (23.9)

*isolates either in blood or urine. APACHE, Acute Physiology and Chronic Health Evaluation.

### Cytokine production *ex vivo*

**Figure 1:**
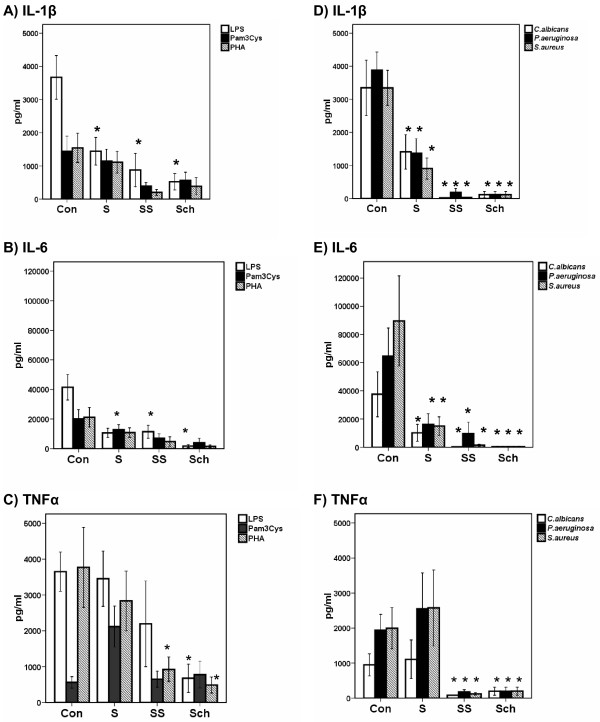
**Release of pro-inflammatory cytokines by PBMCs of healthy controls (Con, *n *= 14) and of septic patients**. Patients are classified as sepsis (S, *n *= 14), severe sepsis (SS, *n *= 18) and septic shock (Sch, *n *= 11). Cells were stimulated with 10 ng/ml of lipopolysaccharide of *Escherichia coli *O55:H5 (LPS); with 5 μg/ml of Pam3Cys; and with 5 μg/ml of phytohemmaglutin (PHA) (**A, B **and **C**); and with 5 × 10^5 ^cfu/ml of heat-inactivated isolates of *Candida albicans*, of multidrug-resistant *Pseudomonas aeruginosa *and of methicillin-resistant *Staphylococcus aureus *(**D, E **and **F**). Asterisks denote statistically significant differences compared with the respective healthy controls.

The concentration of pro-inflammatory cytokines in supernatants of PBMCs isolated from septic patients after stimulation with the various bacterial components was significantly reduced compared to healthy controls (Figure 1). The severity of sepsis was reflected by the degree of cytokine production: PBMCs isolated from patients with septic shock produced less cytokines than those of patients with severe sepsis, which in turn produced less than those from patients with uncomplicated sepsis. Production of IL-1β and of IL-6 was impaired after stimulation with LPS and with heat-killed bacteria but not after stimulation with Pam3Cys and PHA. This was found for all disease stages (panels A, B, D and E). Down-regulation of IL-1β and of IL-6 followed a different pattern than TNFα; release of TNFα was not impaired after LPS stimulation of PBMCs of patients with uncomplicated sepsis and with severe sepsis; moreover after stimulation with heat-killed bacteria TNFα production in uncomplicated sepsis did not differ from controls (panels C and F). IL-10 in supernatants of LPS-stimulated PBMCs from 14 patients with uncomplicated sepsis, 12 patients with severe sepsis and 18 patients with septic shock was below the limit of detection (data not shown), showing that the release of IL-10 did not differ within the stages of sepsis in a similar way as pro-inflammatory cytokines differed.

These findings led us to hypothesize that inhibition of *ex vivo *cytokine release by PBMCs in clinical sepsis is modulated in different ways for IL-1β and for TNFα after stimulation with LPS. This is supported by the finding that the release of IL-6 followed IL-1β, as expected [6].

**Figure 2:**
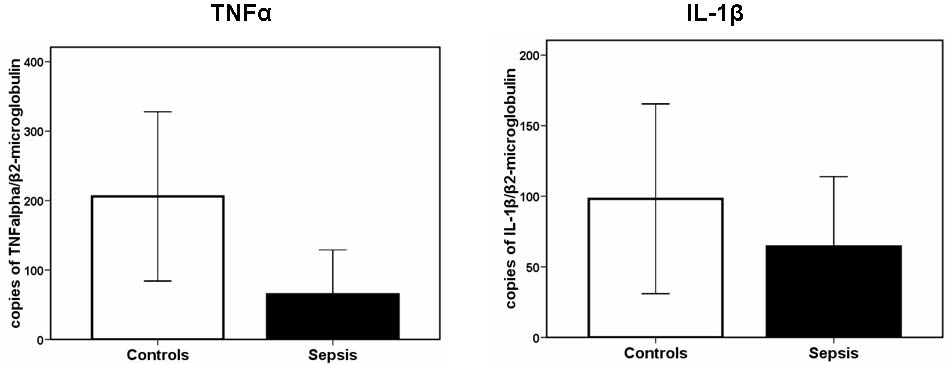
**mRNA transcripts after stimulation of PBMCs of four healthy controls and of six patients with sepsis syndrome**. Cells were stimulated with 10 ng/ml of lipopolysaccharide of *Escherichia coli *O55:H5.

One explanation for the reduced production of cytokines during sepsis is that transcription of proinflammatory cytokines is reduced. Therefore, the number of RNA transcripts of TNFα and of IL-1β in the cell lysates of PBMCs of four healthy volunteers and of six septic patients was determined (Figure 2). Although transcripts of PBMCs of septic patients were lower in the case of TNFα, they only showed a moderate decrease of IL-1β mRNA compared to healthy controls, and this difference was not statistically significant. It is therefore tempting to hypothesize that additional mechanisms are involved in the inhibition.

### Caspase-1 in sepsis and after LPS infusion

**Figure 3:**
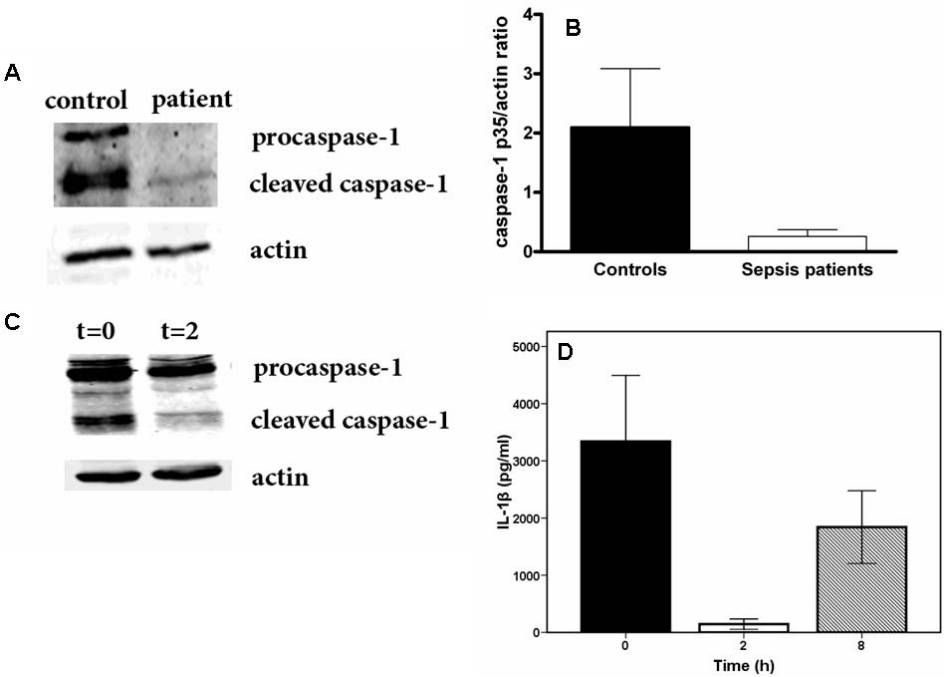
**Western blots of lysates of PBMCs of patients with sepsis and of volunteers with endotoxemia**. Blots show that cleaved caspase-1 is lost in sepsis. **(A) **one healthy control and one patient with sepsis (data representative of five sepsis patients tested); **(B) **quantitative assessment of blots of lysates of PBMCs for four healthy volunteers and for five patients with sepsis; **(C) **Western blots of caspase-1 before and two hours after *in vivo *LPS infusion in one healthy volunteer (representative of four volunteers). **(D) **IL-1β production by LPS-stimulated PBMCs isolated at t = 0, t = 2 and t = 8 hours after LPS infusion in healthy volunteers.

IL-1β is not only regulated at the level of transcription, but also at the level of processing of pro-IL-1β by caspase-1 [6]. Therefore, we performed Western blot analysis of caspase-1. As shown by the Western blot presented in Figure 3A, B, the amount of both pro-caspase-1 and caspase-1 is diminished in sepsis. Since sepsis in this series of patients was mainly caused by Gram-negative bacteria (Table 1), we also investigated caspase-1 activity in volunteers injected intravenously with LPS. As depicted in Figure 3C, caspase-1 activation was markedly decreased in cell lysates of these volunteers. The decrease in caspase-1 was accompanied by near complete absence of IL-1β production by PBMCs stimulated *ex vivo *with LPS. The effect of LPS infusion on IL-1β production was partially restored eight hours after the infusion (Figure 3D).

**Figure 4:**
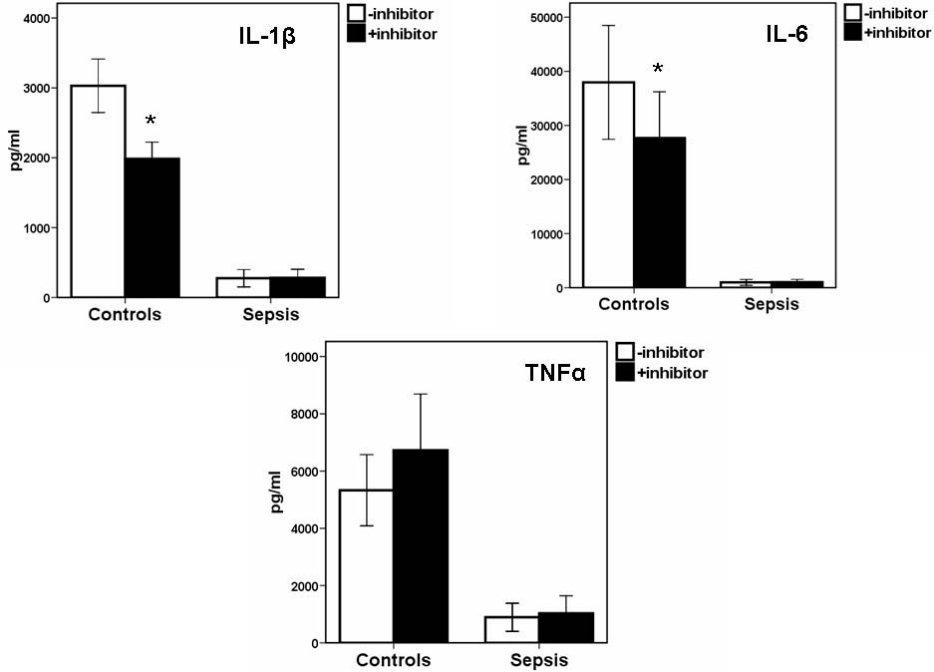
**Release of pro-inflammatory cytokines from PBMCs of 13 healthy controls and of 40 septic patients**. Patients with sepsis (*n *= 20,) with severe sepsis (*n *= 14) and septic shock (*n *= 6) are encountered together. Cells were stimulated with 10 ng/ml of lipopolysaccharide of *Escherichia coli *O55:H5 in the absence or presence of caspase-1 inhibitor. Asterisks denote statistically significant differences of respective comparisons in the absence of inhibitor.

**Figure 5:**
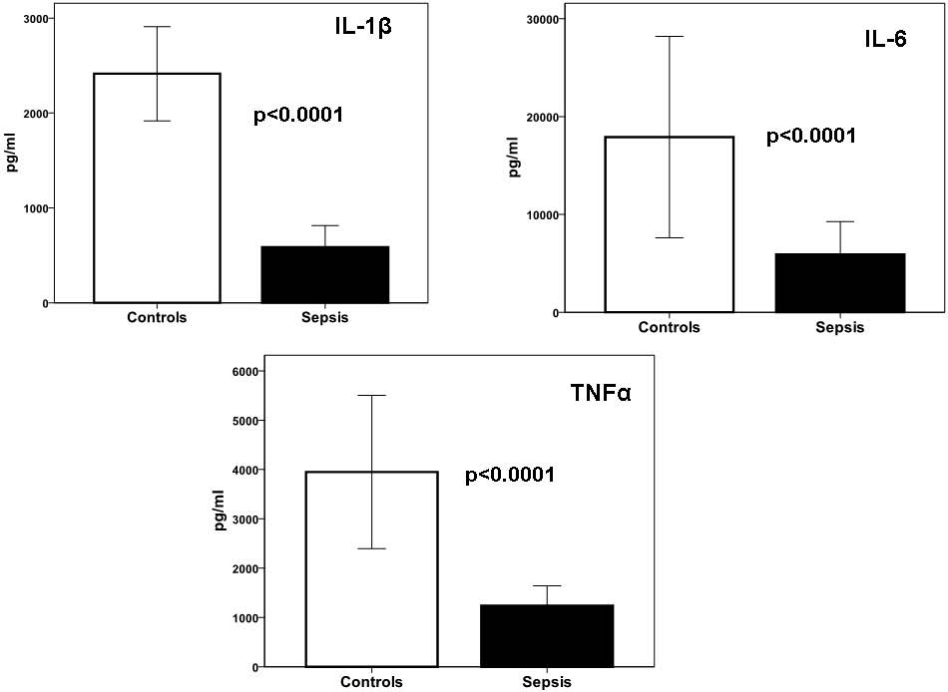
**Release of pro-inflammatory cytokines from PBMCs of 13 healthy controls and of 40 septic patients**. Patients with sepsis (*n *= 20), with severe sepsis (*n *= 14) and with septic shock (*n *= 6) are encountered together. Cells were stimulated with 100 μg/ml of monosodium urate (MSU) in the presence of 10 ng/ml of lipopolysaccharide of *Escherichia coli *O55:H5 (LPS). *P *signifies statistical differences between patients.

**Figure 6:**
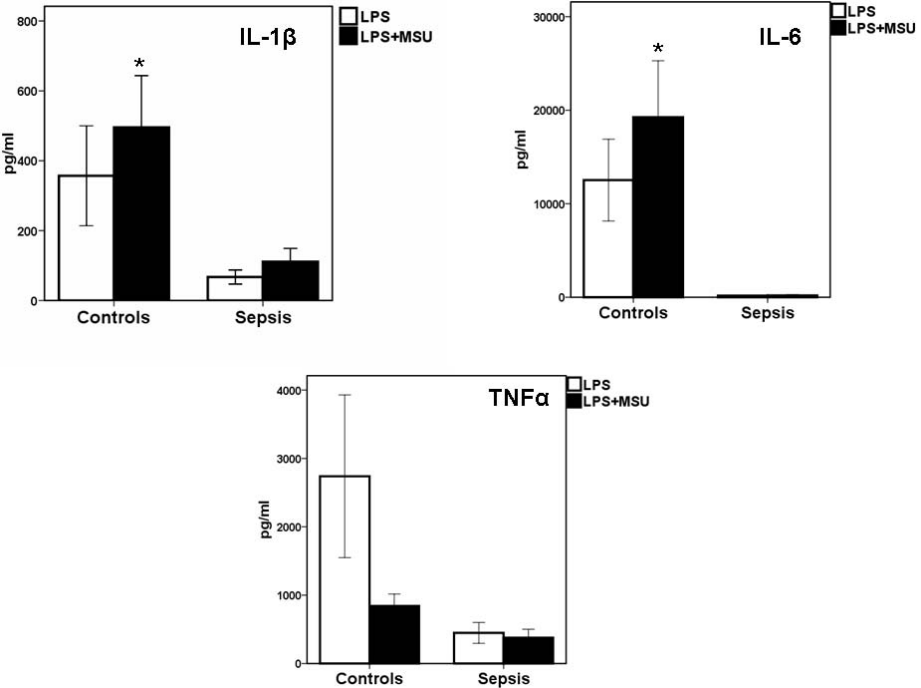
**Release of pro-inflammatory cytokines from PBMCs of 10 healthy controls and of 25 septic patients**. Patients with sepsis (*n *= 12) with severe sepsis (*n *= 10) and with septic shock (*n *= 3) are encountered together. Cells were stimulated with single 0.1 ng/ml of lipopolysaccharide of *Escherichia coli *O55:H5 (LPS) or its interaction with 10 μg/ml of monosodium urate (MSU). Asterisks denote statistically significant differences compared with single LPS.

If the reduced caspase-1 activity is responsible for the decreased production of IL-1β, blocking caspase-1 in septic patients would have limited or no effect. Indeed, caspase-1 inhibition with the caspase-1 inhibitor YVAD had no effects on IL-1β production when PBMCs isolated from patients with sepsis were stimulated with LPS (Figure 4). Monosodium urate (MSU) is able to activate the NLPR3 inflammasome, resulting in caspase-1 activation [7]. To investigate whether NLPR3-stimulated activation of caspase-1 activation was impaired, we used MSU as a stimulus. Stimulation with MSU in the presence of LPS resulted in release of IL-1β from PBMCs isolated from healthy controls, but not from PBMCs of patients with sepsis (Figure 5). We have previously observed that LPS at very low concentrations of 0.1 ng/ml synergizes with MSU (0.1 ng/ml) to induce excess release of IL-1β but not of TNFα [15]. Although this synergy was depicted here in healthy volunteers, it was lost in sepsis patients (Figure 6).

## Discussion

In this study of patients with sepsis and of human volunteers exposed to intravenous endotoxin, we show that production of cytokines *ex vivo *is generally down-regulated proportionally to the severity of sepsis. The pattern of production of IL-1β is different from that with TNFα, as IL-1β is being down-regulated both in severe sepsis and in uncomplicated sepsis, while TNFα production is sustained in this last category of patients. Because of the only moderate decrease in IL-1β mRNA, we hypothesized that single inhibition of transcription cannot explain the decreased IL-1β production and the differential regulation of TNFα and IL-1β. This led us to investigate caspase-1 activity. We demonstrated that active caspase-1 which cleaves pro-IL-1β into bioactive IL-1β, was nearly absent in patients with sepsis or in volunteers receiving an LPS infusion. In line with that, stimulation of the NLPR3 inflammasome by uric acid crystals was significantly impaired in patients with sepsis. Part of the impaired activation of the NLPR3 inflammasome may be due to the very low amount of procaspase-1 seen in sepsis patients. However, the amount of procaspase-1 was less affected in experimental endotoxemia probably showing that conversion of procaspase-1 to caspase-1 was affected.

Down regulation of *ex vivo *cytokine production is well documented in patients with sepsis [16-18], in patients with severe infections [19] and after experimental endotoxin challenge [12]. This phenomenon has been given several names in the literature, although it is not entirely clear whether it regards the same process. The older literature describes it as endotoxin tolerance; another name is immunoparalysis. In the description of the latter, a lot of weight is given to impaired T-cell dependent adaptive immune responses associated with decreased expression of MHC class II, decreased lymphocyte proliferation and T-cell apoptosis [20-22], but the pathophysiological relevance of these features is unclear. Previous studies have reported down-regulation of cytokine production from monocytes of septic patients after stimulation with selective TLR agonists [16-19]. We demonstrate here that the down-regulation occurs irrespective of the stimulant, being either microbes or microbial components. Impaired cytokine production after stimulation with heat-killed whole microorganisms is described for the first time, to our knowledge, and it is of considerable pathophysiological significance for four reasons: a) heat-killed whole microorganisms contain a broad panel of PAMPs; b) the applied microorganisms are blood isolates from septic patients; c) impairment of cytokine response is indicative of a predisposition of the septic host for super-infections; and d) many clinical trials have been conducted with the application of agents aiming to suppress the over-activity of the pro-inflammatory cascade in sepsis [2]. The present results denote that during the clinical course of sepsis the opposite phenomenon occurs with down-regulation of the release of pro-inflammatory cytokines by circulating monocytes and may explain, at least in part, the failure of most of these trials.

Down-regulation of cytokine production was accompanied by reduced transcription of pro-inflammatory cytokines as a mechanism underlying the decreased cytokine production, confirming findings of others [16,17]. As already described elsewhere [19], inhibition of transcription was moderate for IL-1β mRNA whereas production was inhibited up to 90%, suggesting that additional mechanisms are involved. As mentioned above, we found that caspase-1 activation was decreased in sepsis and after endotoxin challenge. This, together with the lesser transcription of IL-1β, may well explain its decreased production. Our finding regarding caspase-1 protein are in accordance with a recent report showing that mRNA expression of the inflammasome components ASC and caspase-1 is reduced in monocytes of patients with septic shock [23]. Caspase-1 activation is constitutively present in human primary monocytes isolated from healthy volunteers [24], yet it is absent in patients with sepsis syndrome, as shown in the present study.

One would expect that microbial components in sepsis trigger the inflammasome, at least initially. In contrast, the activation of the inflammasome ceases pretty soon after the onset of sepsis. It is noteworthy that already two hours after LPS infusion down-regulation of caspase-1 occurs. At that stage, we document decreased conversion from pro-caspase-1 to caspase-1, and the timeframe may preclude a notable effect on transcription and translation of pro-caspase-1. The immunoblots of the sepsis patients, however, are compatible with decreased transcription and translation of caspase-1, as reported by Fahy *et al*. [23]. In addition, we found that white blood cells of septic patients did not respond properly to urate crystals, a trigger of the NLPR3 inflammasome. The observed decreased of IL-1β production may also explain why IL-6 followed similar kinetics whereas TNFα does not since IL-1β regulates production of IL-6 [6].

Whether the decreased caspase-1 activity is a beneficial compensatory mechanism is currently unclear. On the one hand, in experimental models caspase-1 activation contributes to mortality during Gram-negative sepsis, as caspase-1 deficient mice are protected against LPS-induced systemic inflammation and *E. coli*-induced lethal peritonitis [25,26]. On the other hand, caspase-1 activation of IL-1β and IL-18 also represents protective host defense mechanisms, and their inactivation may well be an important component of immunoparalysis in sepsis.

## Conclusions

This study documenting decreased activation of caspase-1 and inhibited cytokine responses in septic patients and in volunteers exposed to intravenous endotoxin provides new insights into the mechanism of cytokine down-regulation in sepsis patients. Further investigations are needed to assess whether these findings can be exploited in therapeutic interventions.

## Key messages

• Blood monocytes of patients with sepsis are characterized by impaired release of pro-inflammatory cytokines after *ex vivo *stimulation. This impairment is related with disease severity and it is particularly pronounced for IL-1β.

• Defective *ex vivo *release of IL-1β is related not only with reduced gene transcription but also with reduced activation of the inflammasome.

## Abbreviations

CFU: colony-forming units; DMSO: dimethyl sulfoxide; HIV: human immunodeficiency virus; IL-1β: interleukin-1beta; IL-6: interleukin-6; LPS: lipopolysaccharide; MDP: muramyldipeptide; MSU: monosodium urate; PBMCs: peripheral blood mononuclear cells; PBS: phosphate buffered saline; PCR: polymerase chain reaction; PHA: phytohemagglutin; PRRs: pattern recognition receptors; TNFα: tumour necrosis factor-alpha; WBCs: white blood cells.

## Competing interests

The authors declare that they have no competing interests.

## Authors' contributions

EJGB designed the study in patients with sepsis, performed statistical analysis, and wrote the manuscript. FLV performed cell stimulation and Western blot analysis in experimental endotoxemia and Western blot analysis in human sepsis, and drafted the manuscript. MM performed quantitative PCR analysis, cell stimulations in patients with sepsis, and drafted the manuscript. MR, AA and AS collected clinical data, and drafted the manuscript. LABJ designed the study of human endotoxemia, and drafted the manuscript. PP conducted the experiments of experimental endotoxemia, and drafted the manuscript. MG performed cytokine measurements and drafted the manuscript. JWMM and MGN designed the study of human sepsis, and drafted the manuscript. All authors read and approved the final manuscript.
